# A 2-year retrospective analysis of the prognostic value of MqSOFA compared to lactate, NEWS and qSOFA in patients with sepsis

**DOI:** 10.1007/s15010-022-01768-0

**Published:** 2022-02-18

**Authors:** Matteo Guarino, Benedetta Perna, Alfredo De Giorgi, Edoardo Gambuti, Franco Alfano, Elisa Maria Catanese, Stefano Volpato, Andrea Strada, Giacomo Caio, Carlo Contini, Roberto De Giorgio

**Affiliations:** 1 Department of Translational Medicine, Internal Medicine Unit, St. Anna University Hospital of Ferrara, University of Ferrara, Via A. Moro 8, Cona, 44124 Ferrara, Italy; 2 Department of Internal Medicine, St. Anna University Hospital of Ferrara, Ferrara, Italy; 3 Department of Medical Sciences, St. Anna University Hospital of Ferrara, University of Ferrara, Ferrara, Italy; 4 Department of Emergency Medicine, St. Anna University Hospital of Ferrara, Ferrara, Italy; 5 Infectious and Dermatology Diseases, St. Anna University Hospital of Ferrara, University of Ferrara, Ferrara, Italy

**Keywords:** In-hospital mortality, Lactate, MqSOFA, NEWS, qSOFA, Sepsis

## Abstract

**Purpose:**

Sepsis is a life-threating organ dysfunction caused by a dysregulated host response to infection. Being a time-dependent condition, the present study aims to compare a recently established score, i.e., modified quick SOFA (MqSOFA), with other existing tools commonly applied to predict in-hospital mortality.

**Methods:**

All cases of sepsis and septic shock consecutively observed at St. Anna University Hospital of Ferrara, Italy, from January 2017 to December 2018 were included in this study. Each patient was evaluated with MqSOFA, lactate assay, NEWS and qSOFA. Accurate statistical and logistic regression analyses were applied to our database.

**Results:**

A total of 1001 consecutive patients with sepsis/septic shock were retrieved. Among them, 444 were excluded for incomplete details about vital parameters; thus, 556 patients were eligible for the study. Data analysis showed that MqSOFA, NEWS and lactate assay provided a better predictive ability than qSOFA in terms of in-hospital mortality (*p* < 0.001). Aetiology-based stratification in 5 subgroups demonstrated the superiority of NEWS vs. other tools in predicting fatal outcomes (*p* = 0.030 respiratory, *p* = 0.036 urinary, *p* = 0.044 abdominal, *p* = 0.047 miscellaneous and *p* = 0.041 for indeterminate causes). After Bonferroni’s correction, MqSOFA was superior to qSOFA over respiratory (*p* < 0.001) and urinary (*p* < 0.001) aetiologies. Age was an independent factor for negative outcomes (*p* < 0.001).

**Conclusions:**

MqSOFA, NEWS and lactate assay better predicted in-hospital mortality compared to qSOFA. Since sepsis needs a time-dependent assessment, an easier and non-invasive score, i.e., MqSOFA, could be used to establish patients’ outcome in the emergency setting.

## Introduction

Sepsis is a life-threating organ dysfunction resulting from a dysregulated host response to wide range of infections [1, 2]. Its incidence is 4 per 1000 people in the Italian population with a steadily increasing mortality rate in the last 15 years [3]. Despite treatment advances, septic patients have a high risk of in-hospital mortality (IHM), reaching 20% or more in some settings, making sepsis and septic shock one of the highest mortality conditions in the Emergency Department (ED) [4–6]. Diagnostic criteria were defined during the third international consensus on sepsis and septic shock (Sepsis-3), when a pool of experts reached a consensus on quick sequential organ failure assessment (qSOFA) and sequential organ failure assessment (SOFA) score to establish the overall organ dysfunction and the risk of mortality for septic patients [1]. Following the indication of Sepsis-3, patients scoring positive for qSOFA (i.e., ≥ 2) should be considered at high risk for sepsis. Subsequently, if SOFA is ≥ 2 a diagnosis of sepsis can be established, whereas septic shock is defined by a more severe clinical picture with hyperlactatemia and severe hypotension requiring a vasopressor (e.g., norepinephrine) to maintain mean arterial pressure ≥ 65 mmHg [1, 6]. These scores helped to identify septic patients better than previous criteria, such as systemic inflammatory response symptoms (SIRS) [6, 7]. As previously reported in our previous study [8], some authors raised concern about the prognostic value of the qSOFA and SOFA in terms of mortality [9–17], thus proposing new predicting scores [8, 17–21] or laboratory tests [22–27] to assess the risk of IHM in septic patients. In our previous study [8], we developed a modified version of qSOFA (MqSOFA) by adding SpO2/FiO2 ratio to the previous score criteria. We showed that the created tool effectively predicted IHM in patients with sepsis. Thus, the primary endpoint of this new study was to compare MqSOFA with different scores/tests, i.e., National Early Warning Score (NEWS), qSOFA (these scores are described in Table 1) and lactate assay to predict the overall risk of IHM.

**Table 1:** Comparison between qSOFA, MqSOFA and NEWS

qSOFA		MqSOFA		
Parameter	Points	Parameter		Points
Blood pressure ≤ 100 mmHg	1	Blood pressure ≤ 100 mmHg		1
Respiratory rate ≥ 22/min	1	Respiratory rate ≥ 22/min		1
Altered mentation	1	Altered mentation		1
		SpO2/FiO2 ratio	≥ 316	0
			236–315	1
			≤ 235	2

NEWS							
Parameter	3	2	1	0	1	2	3
Respiratory rate	≤ 8		9–11	12–20		21–24	≥ 25
O_2_ saturation (%)	≤ 91	92–93	94–95	≥ 96			
Supplemental O_2_		Yes		No			
Temperature (°C)	≤ 35.0		35.1–36.0	36.1–38.0	38.1–39.0	≥ 39.1	
Systolic blood pressure (mmHg)	≤ 90	91–100	101–110	111–219			≥ 220
Heart rate	≤ 40		41–50	51–90	91–110	111–130	≥ 131
Level of consciousness (AVPU)				Alert			Verbal, pain, unresponsive

Red values are those significant for p <0.05. In the pairwaise comparison, we initially considered significant values with p <0.05 and then performed the Bonferroni's correction

Furthermore, as secondary aim, this study proposed the analysis of sepsis aetiology via a stratification in five different groups (i.e., respiratory, urinary, abdominal, miscellaneous and indeterminate infections) and pairwise compared the investigated tools in terms of IHM over each aetiology.

## Materials and methods

In this retrospective, single centre study, all included patients were identified by searching for diagnosis of ‘sepsis’ and ‘septic shock’ in the discharge letter provided by the Emergency Department of St. Anna Hospital, Cona, Ferrara, Italy, from January 2017 to December 2018. We retrieved a total number of 1001 individual records; of this, 556 had chart full information available to retrospectively valuate the level of blood lactates and calculate NEWS, qSOFA and MqSOFA scores. For each of the investigated tool, it has been proposed a “high risk” class, identifying patients with a potentially worse outcome, i.e., IHM (MqSOFA ≥ 2, lactates ≥ 1.85, NEWS ≥ 7 and qSOFA ≥ 2). Intubated patients were not recruited in this study.

### Statistical analysis

Categorical data were expressed as absolute frequencies and percentages, while means ± standard deviation (SD) were reported for continuous variables. Differences between patients deceased or discharged for sepsis were compared with the Pearson’s *X*^2^, student *t* tests and Mann–Whitney tests as appropriate. The association between IHM and the investigated tools (i.e., NEWS, qSOFA, MqSOFA and the lactate assay) was studied with univariated and multivariated logistic regression analysis. Odds ratios (ORs) and their 95% confidence intervals (CI) were reported. Moreover, the areas under the curve (AUC) of the receiver operating characteristic (ROC) curves were pairwise compared to identify the tool with the best discriminative ability. Because of lactates showed a non-normal distribution, the natural logarithm of lactates was calculated to obtain ORs. Moreover, similar analysis with ROC curves were performed with five different groups of sepsis aetiologies, such as respiratory, urinary, abdominal, miscellaneous and indeterminate.

The Statistical Product and Service Solution (SPSS) 23.0 for Windows (IBM Corp., Armonk, NY, USA) and MedCalc^®^ Statistical Software version 19.8 (MedCalc Software Ltd, Ostend, Belgium) were used for statistical analyses and the significance level was set for *p* < 0.05.

## Results

A total number of 1001 consecutive cases of sepsis and septic shock were retrieved. Among them 445 were excluded for incomplete details about vital parameters or lactate levels; thus, 556 patients were eligible for the study; of these, 253 were males (45.5%) and 303 were female (54.5%) with a mean age of 79.9 ± 11.9 years (19–99 years). A total number of 338 patients (60.8%) were discharged, whereas 218 (39.2%) died because of sepsis. No statistically significant differences in terms of IHM between male and female (*p* = 0.384) were found. In the subset with fatal outcome, age was significantly higher in the subgroup of deceased vs. discharged patients (82.5 ± 10.9 years vs. 78.2 ± 12.3 years; *p* < 0.001) resulting in a negatively discriminant factor (OR 1.02, 95% C.I. 1.00–1.04; *p* = 0.044). The OR for one-unit increase in the score was found to be greater for lactates (OR 5.020) than for MqSOFA, which associated with lower OR values of 2.56; the results were consistent after age adjustment (Table 2).

**Table 2:** Logistic regression analysis of in-hospital mortality

	Univariate model			Multivariate/age-adjusted model		
	OR	95% CI	*p*	OR	95% CI	*p*
MqSOFA	2.560	(2.183–3.002)	< 0.001	2.522	(2.149–2.960)	< 0.001
Lactate	5.017	(3.766–6-683)	< 0.001	4.933	(3.692–6.590)	< 0.001
NEWS	3.273	(2.652–4.039)	< 0.001	3.206	(2.592–3.965)	< 0.001
qSOFA	2.583	(2.101–3.176)	< 0.001	2.507	(2.035–3.090)	< 0.001

**Fig. 1:**
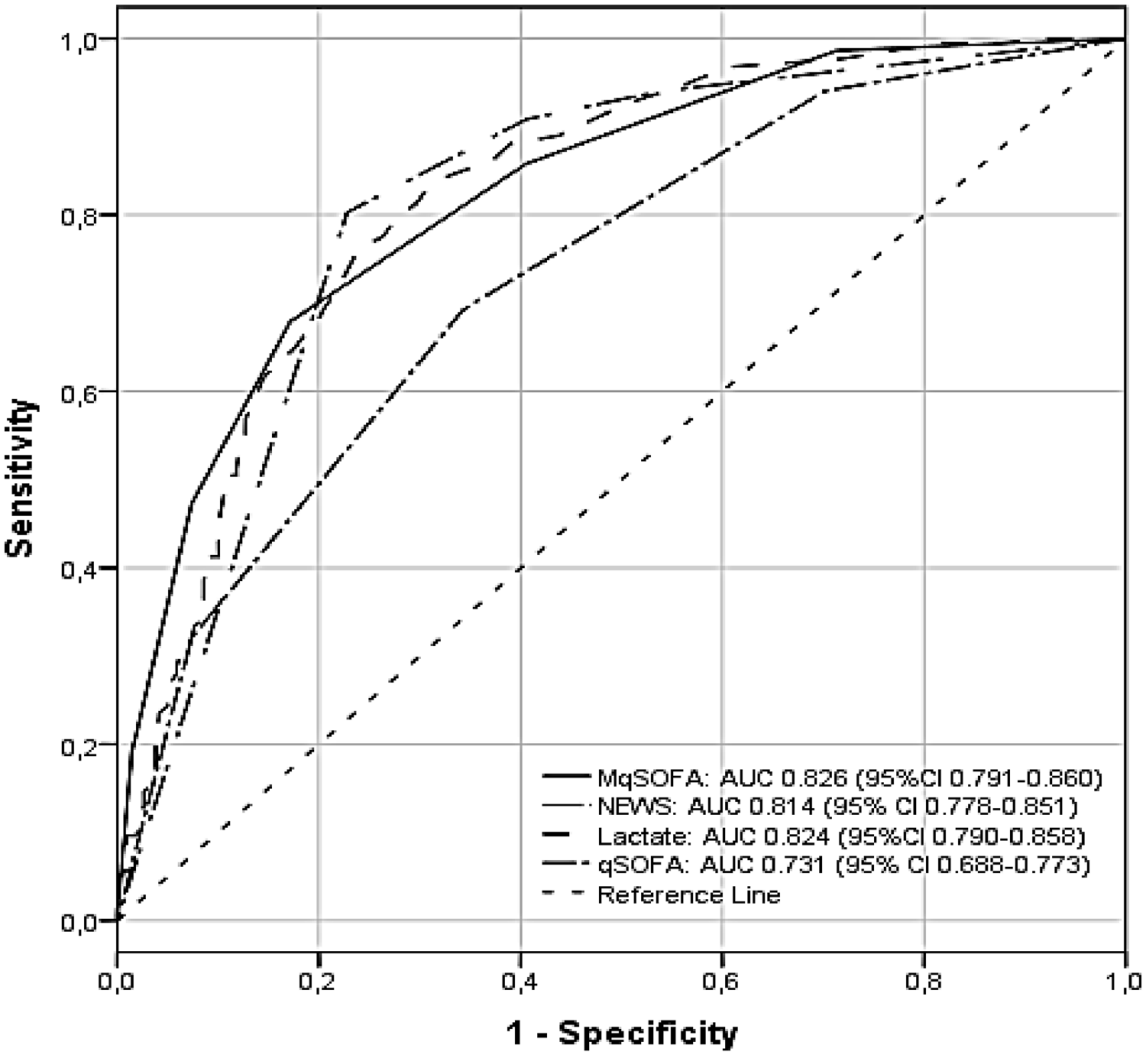
Comparison of ROC curves of qSOFA, MqSOFA, NEWS and lactate assay to assess in-hospital mortality

Figure 1 shows no statistically significant difference among the ROCs of NEWS, MqSOFA and lactate assay, highlighting the superiority of these three tools over qSOFA (*p* < 0.001) in predicting IHM. Since each of the involved tools has a “high risk” level (i.e., MqSOFA ≥ 2, lactates ≥ 1.85, NEWS ≥ 7 and qSOFA ≥ 2), Table 3 shows percentage values of sensitivity, specificity, accuracy, positive predictive value (PPV) and negative predictive value (NPV) related to this class of risk.

**Table 3:** Levels (%) of sensitivity, specificity, accuracy, positive predictive value (PPV) and negative predictive value (NPV) for “high-risk” stratification of each score

	Sensitivity (%)	Specificity (%)	Accuracy (%)	PPV (%)	NPV (%)
MqSOFA ≥ 2	85.8	59.9	69.8	57.7	86.6
Lactates ≥ 1.85	83.5	68.9	74.6	63.4	86.6
NEWS ≥ 7	80.7	77.2	78.6	69.6	86.1
qSOFA ≥ 2	69.3	65.7	67.1	56.6	76.8

Sepsis aetiologies have been categorized in five different groups, i.e., respiratory (*n* = 188, 33.8%), urinary (*n* = 180, 32.4%), abdominal (*n* = 67, 12.1%), miscellaneous (*n* = 48, 8.6%) and indeterminate (*n* = 73, 13.1%). The univariate analysis showed that sepsis due to indeterminate cause was related to the highest IHM (respiratory 43.1%, urinary 31.1%, abdominal 35.8%, miscellaneous 27.1% and indeterminate 60.3%). Table 4 shows the AUCs of analysed scores over different aetiologies and the pairwise comparison of the curves among each other. NEWS was the only score which reached statistical significance regardless the aetiology (*p* = 0.030 for respiratory, *p* = 0.036 for urinary, *p* = 0.044 for abdominal, *p* = 0.047 for miscellaneous and *p* = 0.041 for indeterminate infections) although MqSOFA, lactate assay and qSOFA were close to significance. In the pairwise comparison of AUCs, MqSOFA was the only score superior to qSOFA over each aetiology (*p* < 0.001 for respiratory, *p* < 0.001 for urinary, *p* = 0.047 for abdominal, *p* = 0.021 for miscellaneous and *p* = 0.024 for indeterminate infections). After Bonferroni’s correction, MqSOFA was still superior to qSOFA for respiratory and urinary causes.

**Table 4:** AUC confrontation between MqSOFA, lactate assay, NEWS and qSOFA in terms of in-hospital mortality over aetiology and pairwise comparison of ROC curves

		AUC	95% CI	*p*	Pairwise comparison of ROC curves *p*			
					MqSOFA	Lactate	NEWS	qSOFA
Respiratory	MqSOFA	0.832	0.771–0.883	**0.029**		0.993	0.066	< 0.001*
	Lactate	0.832	0.771–0.883	**0.029**	0.993		0.186	0.004*
	NEWS	0.781	0.715–0.838	**0.030**	0.066	0.186		0.054
	qSOFA	0.723	0.635–0.785	**0.036**	< 0.001*	0.004*	0.054	
Urinary	MqSOFA	0.828	0.765–0.880	**0.032**		0.450	**0.015**	< 0.001*
	Lactate	0.796	0.730–0.852	**0.034**	0.450		0.632	0.201
	NEWS	0.774	0.706–0.833	**0.036**	**0.015**	0.632		0.198
	qSOFA	0.735	0.664–0.798	**0.039**	< 0.001*	0.201	0.198	
Abdominal	MqSOFA	0.844	0.734–0.921	**0.047**		0.396	0.587	**0.047**
	Lactate	0.794	0.677–0.883	0.055	0.396		0.231	0.830
	NEWS	0.863	0.758–0.935	**0.044**	0.587	0.231		0.062
	qSOFA	0.781	0.663–0.872	0.054	**0.047**	0.830	0.062	
Miscellaneous	MqSOFA	0.848	0.715–0.935	0.056		0.988	0.054	**0.021**
	Lactate	0.849	0.717–0.936	0.056	0.988		0.701	0.252
	NEWS	0.877	0.750–0.954	**0.047**	0.054	0.701		**0.018**
	qSOFA	0.757	0.612–0.869	0.065	**0.021**	0.252	**0.018**	
Indeterminate	MqSOFA	0.800	0.690–0.885	0.055		0.357	**0.031**	**0.024**
	Lactate	0.865	0.765–0.934	**0.049**	0.357		0.717	0.040
	NEWS	0.887	0.791–0.949	**0.041**	**0.031**	0.717		0.001*
	qSOFA	0.715	0.597–0.815	0.060	**0.024**	**0.040**	0.001*	

^*^New α-level for significance of *p* value was < 0.0042 after Bonferroni’s Correction

## Discussion

Sepsis is an insidious and life-threatening condition that requires a timely diagnosis and treatment based on standardized screening tools. Although often challenging, the early identification of septic patients is mandatory to improve survival [1–6, 28]. However, there is no validated, evidence-based tool or strategy to reliably accomplish this goal in any emergency setting (i.e., ED or out-of-hospital) [7]. The main objective of this article was to compare different screening tools (i.e., MqSOFA, NEWS, and lactate testing) with qSOFA to identify the best performing one.

As reported in a previous article [8], some authors raised concern about Sepsis-3 diagnostic sequence and qSOFA ability to predict IHM [9–17]. Furthermore, among non-invasive tools, NEWS and MqSOFA showed a better prediction of fatal outcome over qSOFA [8, 18]. Advantages and limits of qSOFA and MqSOFA were previously reported [8, 30]; however, an appraisal of the other tools is necessary to fully understand our new results and to establish the clinical relevance of MqSOFA in the emergency settings.

NEWS is a score based on multiple non-invasive parameters, as described in Table 1 [18]. A score from 0 to 3 is assigned for each parameter and the total identifies four classes of risk (0–4 low-risk, a single parameter with 3 points describes a low-medium risk, 5–6 medium risk and ≥ 7 points high risk of fatal outcome). This simplified categorization allowed us to perform an adequate comparison (otherwise difficult because of NEWS complexity) among involved scores. Furthermore, the parameter describing the state of consciousness is based on the alert–voice–pain–unresponsive (AVPU) system, in contrast to qSOFA and MqSOFA, both using the acute alteration of mental status. This slight difference is actually crucial, because in the elderly, the level of consciousness may be chronically altered. This clinical scenario would lead physicians using NEWS to assign 3 points to this parameter, and therefore, the investigated patient would fall at least into a low–intermediate class of risk. In contrast, qSOFA and MqSOFA distinguish between chronic vs. acute cognitive impairment avoiding the overestimation of patients’ conditions. This concept finds support by two ROC curves extrapolated by assessing NEWS in patients with age ≤ 65 and ≥ 80 years. The AUC of NEWS in patients ≤ 65 years is significantly higher than in ≥ 80 years (0.859, 95% CI 0.744–0.974, *p* < 0.001 vs. 0.790, 95% CI 0.741–0.839, *p* < 0.001). This finding highlights that the neurological status can modify the predictive power of this score, which was more specific in younger patients, usually not suffering from chronic cognitive impairment.

Lactate elevation is known to correlate with a higher risk of short-term and long-term mortality [22–24]. Indeed, the study by Liu et al. showed that the lactate assay alone had a superior prognostic accuracy for short-term and long-term mortality than any other criteria, including qSOFA [22]. In contrast to the other scores evaluated in this paper, lactate assay is an invasive laboratory analysis. Despite its high prognostic accuracy, so far this test is not available in out-of-hospital emergency setting. Moreover, considering the mean time of waiting for a medical visit at the ED for patients with suspected infection (about 50 min in our Hospital, which is almost out of the “golden-hour”) a delay in recognizing critical conditions may impact negatively on patients’ survival. In this paper, according Liu et al. [22], lactate assay alone, likewise MqSOFA and NEWS, predicted IHM better than qSOFA. According to ROC curves, the lactate level with the best sensitivity/specificity ratio was 1.85 mg/dl. In particular, a lactate level < 1.85 mg/dl is associated with a 13.4% risk of IHM, whereas ≥ 1.85 increases the risk to 63.4% (sensitivity 83.5%, specificity 68.9%, PPV 63.4%, NPV 86.6%, accuracy 74.6%). These values of sensitivity and specificity of plasma lactate levels were similar to those proposed for 28-day mortality rate detected by Liu et al. with an optimal cutoff value of 2.99 mmol/L (sensitivity 82.6% and specificity 55%) [31]. The utility of lactates in predicting sepsis mortality could be exploited even better if a point-of-care lactate determination would be obtainable in out-of-hospital emergency setting. This assay would allow an early detection of high-risk patients and treatment of those cases with otherwise underestimated condition. Furthermore, an early identification of high-risk patients, even before admission to the ED, would allow physicians to direct patients towards an intensive setting rather than internal medicine ward. In 2017, a review about point-of-care lactate testing for sepsis in the ED and pre-hospital setting indicated the high-quality evidence supporting the use of this tool in predicting IHM [32]. Only one study involving the out-of-hospital setting showed no superiority of point-of-care lactate testing. However, a low number of enrolled patients (*n* = 59) and unclear inclusion criteria (“critically ill, medical, non-trauma patients”) limited this paper [33].

The three tools were superior to qSOFA in predicting IHM (*p* < 0.001). Comparing the AUCs of MqSOFA, NEWS and lactate assay, the best tool was MqSOFA although there were no statistically significant differences in ability to predict the risk of fatal outcomes (MqSOFA vs. NEWS, *p* = 0.429; MqSOFA vs. lactates, *p* = 0.939). Furthermore, MqSOFA showed the highest levels of sensitivity and NPV despite a low specificity and PPV. To minimize the possibility of underestimating potentially critical patients [34], an appropriate score to the emergency settings should have high sensitivity and NPV levels.

We analysed the differences between discharged vs. deceased patients among the five groups of aetiology, highlighting that sepsis of indeterminate origin has a significantly higher risk of IHM. This finding may be explained by considering the difficulty of initiating an empiric antibiotic treatment without knowing the site of infection. Among the AUCs, only NEWS was statistically significant regardless the aetiology; other tools resulted borderline significant for abdominal, miscellaneous and indeterminate infections. However, these three groups included a small sample size and likely a larger subset would be enough to reach statistical significance. In the pairwise comparison of AUCs, MqSOFA was the only score superior to qSOFA over respiratory and urinary aetiologies indicating the usefulness of S/F ratio as predictor of mortality. Indeed, in our previous paper, we performed ancillary tests introducing a ‘useless parameter’, e.g., ‘gender’, to qSOFA thereby creating an ‘altered’ MqSOFA to show that this extra-item did not improve the AUC as compared to qSOFA [8].

Despite progress in treatment, sepsis and septic shock are still life-threatening, reaching a mortality rate of about 20% in Western Countries [2–6]. In this study, the overall IHM was 39.2%, which is higher than that described in the literature. Since the mean age of involved patients in our study was rather high (79.9 ± 11.9 years), an elevated mortality rate was expected. By selecting patients with a cutoff ≤ 65 years, this rate dropped down to 24%, a finding in line with published data [5]. Furthermore, patients considered in this study often required a high intensity of care, which may explain the high IHM rate. Indeed, consistent with previous published evidence, a recent study on a low-intensity medical cohort of patients with suspected infection at Ferrara University-Hospital showed an IHM of 12.7% [29].

We would like to acknowledge some limitations of our study: first, it is a retrospective analysis with a single-centre database, which considerably reduced the statistical power of this investigation. Second, the S/F ratio has limitations related to the SpO2 parameter and its high variability in different clinical conditions [8, 30]. Other limitations concerned the exclusion of a quite high proportion (almost half) of patients for inadequate data and intubated patients. Furthermore, we considered only a single short-term outcome, i.e., IHM, without extending the analysis to long-term period.

## Conclusion

In this single-centre study we confirmed that MqSOFA, NEWS and lactate assay better predicted IHM vs. qSOFA. MqSOFA resulted to be an easier and non-invasive tool compared to NEWS and lactate assay. Since a timely risk assessment in sepsis is mandatory, these two proprieties, combined with high levels of sensitivity and NPV, give MqSOFA a better performance in the emergency settings. The AUC of MqSOFA was higher than the other tools in terms of overall IHM although no statistically significant differences were observed.

Regarding the secondary outcome, the analysis highlighted that NEWS was the only score superior to the others regardless the underlying aetiology. A larger sample size should improve the statistical power of MqSOFA and lactate assay. Furthermore, MqSOFA is the only tool analysed in this paper that is superior to qSOFA, which is the gold-standard for sepsis initial assessment proposed by the guidelines [1, 6]. Future prospective studies, performed on large cohorts, are awaited to demonstrate the efficacy of a simple and inexpensive score, i.e., MqSOFA, in predicting the outcome of patients with sepsis.

## Data Availability

The data sets generated and/or analysed during the current study are not publicly available due to privacy policy but are available from the corresponding author on reasonable request.

## Abbreviations

AUC: Area under the curve
CI: Confidence intervals
ED: Emergency department
FiO_2_: Inspired fraction of oxygen
ICU: Intensive care unit
IHM: In-hospital mortality
MqSOFA: Modified quick sequential organ failure assessment
NEWS: National early warning score
OR: Odds ratio
qSOFA: Quick sequential organ failure assessment
ROC: Receiver operating characteristic
S/F: SpO_2_/FiO_2_ ratio
SD: Standard deviation
SIRS: Systemic inflammatory response syndrome
SOFA: Sequential organ failure assessment
SpO_2_: Peripheral oxygen saturation
PaO_2_: Arterial partial pressure of oxygen
